# Culturally Responsive Opioid and Other Drug Prevention for American Indian/Alaska Native People: a Comparison of Reservation- and Urban-Based Approaches

**DOI:** 10.1007/s11121-022-01396-y

**Published:** 2022-06-24

**Authors:** Kelli A. Komro, Elizabeth J. D‘Amico, Daniel L. Dickerson, Juli R. Skinner, Carrie L. Johnson, Terrence K. Kominsky, Kathy Etz

**Affiliations:** 1 Department of Behavioral, Social, and Health Education Sciences, Rollins School of Public Health, Emory University, Atlanta, GA USA; 2 RAND Corporation, Santa Monica, CA USA; 3 Integrated Substance Abuse Programs (ISAP), University of California Los Angeles, Los Angeles, CA USA; 4 Cherokee Nation Behavioral Health, Tahlequah, OK USA; 5 Sacred Path Indigenous Wellness Center, San Dimas, CA USA; 6 Epidemiology Research Branch, National Institute On Drug Abuse, North Bethesda, USA

**Keywords:** American Indian/Alaska Native people, Drug prevention, Culturally responsive

## Abstract

There are few substance use treatment and prevention programs for AI/AN people that integrate culturally based practices with evidence-based treatment and prevention. The National Institutes of Health’s (NIH’s) Helping to End Addiction Long-term (HEAL) Prevention Cooperative supports two projects focused on AI/AN populations. One focuses on youth ages 15 to 20 years living within the Cherokee Nation reservation, a multicultural rural area in northeastern Oklahoma, and the second focuses on emerging adults ages 18 to 25 years living in diverse urban areas. We provide a brief overview of the two prevention trials and a case comparison across approaches using the framework of promising practices for intervention science with Indigenous communities (Whitesell et al., 2020) related to (1) integration of Indigenous and academic perspectives to respond to community needs, (2) community partnership and engagement, (3) alignment with Indigenous cultural values and practices, (4) capacity building and empowerment, (5) implementation within complex cultural contexts, and (6) tribal oversight. Overall, these two projects highlight the importance of long-standing relationships with community partners, engaging the community at all levels to ensure that programming is culturally and developmentally appropriate, and having tribal and elder oversight. These practices are key to establishing trust and building confidence in research in these communities and ensuring that research can benefit AI/AN people. These studies showcase how strong partnerships can advance health and support the conduct of rigorous science to help pinpoint optimal health solutions by identifying efficacious, culturally grounded intervention strategies. Although the sovereign status of tribes demands this type of partnership, this research serves as a model for all community research that has a goal of improving health.

## Introduction

American Indian/Alaska Native (AI/AN) tribes and people have built on strengths of their families and cultural practices to address historical and contemporary challenges they face (Cross et al., 2000; Whitbeck et al., 2012). Despite inherent and cultural strengths, AI/AN people have been subjected to hardships, many as a result of government policies, that have negatively affected social determinants of health and increased the prevalence for numerous health conditions (Sequist, 2017). Furthermore, AI/AN individuals’ exposure to historically based traumas, including land dispossession, forced removals, and attempted forced assimilation (Duran & Duran, 1995; Witko, 2006), have been postulated to contribute to intergenerational transmission of health disparities, including substance use disorders.

While most attention to opioid misuse and opioid-involved overdose deaths in the USA has focused on White populations in rural areas, AI/AN populations have opioid overdose deaths at equal or greater levels (Ahmad et al., 2021). Both rural and urban AI/AN populations have been disproportionately burdened by opioid misuse, overdose, and death (Palombi et al., 2018; Wilson et al., 2020; Stanley et al., 2021). From 1999 to 2016, opioid overdose mortality rates among the AI/AN population rose rapidly, continuously, and parallel to rates among non-Hispanic Whites (Tipps et al., 2018). Drug overdose deaths continue to rise (Ahmad et al., 2021), with an urgency for testing effective prevention approaches across the continuum from universal to indicated. In fact, early onset of alcohol and drug use, for which AI/AN youth are at greater risk, increases the likelihood of opioid and other drug use into adulthood (Stanley et al., 2020), putting early onset youth at increased risk. Prevention efforts during this developmental period are critical.

### State of Prevention Science with AI/AN Populations

Despite long-term calls for respecting Indigenous traditional knowledge, cultural practices, and culturally specific prevention (Gone, 2012; Whitbeck, 2006; Whitbeck et al., 2012), there are few substance use prevention programs for AI/AN people that prioritize cultural practices or appropriately design culturally specific strategies with European American evidence-based prevention (Blue Bird Jernigan et al., 2020; Crump et al., 2020, D'Amico, Dickerson et al., 2020, Rasmus, 2014; Whitesell et al., 2020; Snijder et al., 2021). In response to community suggestions for culturally appropriate and resilience-based approaches to prevention in AI/AN communities (Allen et al., 2006; Dickerson et al., 2012; Blue Bird Jernigan et al., 2020; Dickerson et al., 2021; Rasmus et al., 2019), development of culturally centered interventions integrating Indigenous theories and knowledge systems with Western intervention paradigms have gained momentum across tribal populations (Allen et al., 2006; Allen et al., 2018, Blue Bird Jernigan et al., 2020; Dickerson, D’Amico et al., 2021; Dickerson et al., 2021). In 2012, the NIH began a special funding initiative to address limited research in this area (Crump et al., 2020), which led to numerous studies with partnerships between researchers and AI/AN communities to develop and test culturally appropriate interventions for a variety of behaviors (Dickerson et al., 2020).

To address the ongoing opioid crisis, there remain few evidence-based, community-informed, culturally centered prevention interventions for AI/AN people. This is noteworthy, as several recent randomized controlled trials have shown that community and culturally centered preventive interventions can help create effective and sustainable programs for AI/AN populations (Allen et al., 2018; Komro et al., 2017; Livingston et al., 2018; Rasmus et al., 2019; D'Amico et al., 2020; Dickerson et al., 2020; Kaholokula et al., 2021). Thus, it is crucial to continue to encourage traditional AI/AN ideals of wellness and cultural identity to create and sustain culturally centered opioid prevention interventions that incorporate community leadership and empowerment.

Tribal responses to the opioid epidemic among AI/AN people often recognize culture and sovereignty as important elements, providing solutions that include education, prevention, and treatment. Challenges remain for urban populations. A California state-wide needs assessment identified gaps in opioid prevention, treatment, and recovery services targeting AI/AN communities (Soto et al., 2019). Based on interviews and focus groups, the report recommended the provision of culturally centered interventions that integrate traditional approaches to decrease the impact and burden of opioid-related mortality among AI/AN people. Within reservation-based settings, coordinated local solutions are more feasible. The Cherokee Nation Health Services provides a systematic set of prevention, treatment, and recovery services. Efforts include a tribal-wide social norms campaign to raise awareness around opioid use and public education on safe storage and disposal of prescription opioids in home, medical, and community settings. Another example is the Mashpee Wampanoag Tribe dedicating their annual powwow to the opioid epidemic, incorporating an educational component with speakers from the community, treatment facilities, law enforcement, and federal health agencies (Mashpee Wampanoag Tribe, 2021). The sovereignty of tribes means that solutions can be developed across all tribal services, providing an opportunity to coordinate responses in a way that can be more complicated in larger jurisdictions.

### AI/AN Needs in Rural Reservation and Urban Settings

AI/AN reservations are primarily in rural areas, with public health and medical infrastructure, resources, and support for community organizations often limited (Meit & Knudson, 2017). Despite prevention and treatment services provided by the Cherokee Nation, for example, challenges remain in reach given the sheer number of AI people needing behavioral health services within the large and geographically dispersed area of the reservation. Even in the face of new funding opportunities, such as Substance Abuse and Mental Health Services Administration’s (SAMHSA’s) Tribal Opioid Response grants (Substance Abuse & Mental Health Services Administration, 2021), developing solutions remains a challenge. Therefore, it is important to implement culturally appropriate strategies to engage local people and build on their strengths, by providing support, skill building, evidence-based strategies for local solutions, and infrastructure for sustained prevention efforts.

Urban settings also present unique challenges. Although 70% of AI/AN individuals reside in urban areas (Norris, 2012), the relative population of AI/AN people is small in comparison to other racial/ethnic groups. As a result, it is often difficult to engage AI/AN people in prevention and intervention efforts as opportunities to reach out to this population can be challenging due to the complex urban landscape and decreased opportunities to access cultural events and health-promoting activities (D’Amico, Dickerson et al., 2020; D’Amico, Palimaru et al., 2020; Dickerson et al., 2016). Furthermore, numerous federal definitions of what constitutes an AI/AN community and tribal nation typically do not include this large segment of AI/AN people, thus limiting opportunities for larger public health approaches and research to address important health issues that may affect urban dwelling AI/AN individuals.

### HEAL Prevention Initiative

The NIH’s Helping to End Addiction Long-term (HEAL) Prevention Initiative is administered by the National Institute on Drug Abuse (NIDA) and includes a diverse collection of research projects to improve prevention for opioid misuse and addiction. The HEAL Prevention Cooperative (HPC), which is part of the HEAL Prevention Initiative (HPI), includes 10 research projects and one coordinating center that work across a variety of settings and populations to test preventive intervention strategies for adolescents and young adults. Two of the HPC projects focus on AI/AN populations, one focused on youth ages 15 to 20 years living within the Cherokee Nation reservation, a multicultural rural area in northeastern Oklahoma, and the second focused on emerging adults ages 18 to 25 years living in diverse urban areas.

This paper provides a case comparison of the two AI/AN prevention trials and compares approaches in a rural reservation setting to approaches in urban areas. We use the framework of promising practices for intervention science with Indigenous communities (Whitesell et al., 2020) to compare methods and highlight similarities and differences. We describe similar practices that are being implemented across settings, including (1) integration of Indigenous and academic perspectives to respond to community needs, (2) community partnership and engagement, (3) alignment with Indigenous cultural values and practices, and (4) capacity building and empowerment. Given significant differences across reservation and urban settings, we also describe distinct methods across the two sites with regard to (1) implementation within complex cultural contexts and (2) tribal oversight.

## Comparison of Reservation- and Urban-Based Approaches

### Overview of Prevention Trials

#### Trial with Reservation-Based Youth

The prevention trial in the Cherokee Nation is led by public health scientists at Emory University and leadership, evaluation, and clinical team members of Cherokee Nation Behavioral Health (CNBH). CNBH provides treatment and recovery services, as well as community prevention efforts, including long-standing community coalitions funded by SAMHSA prevention grants. The trial received approval from the Cherokee Nation IRB.

The current trial builds upon a previous alcohol use prevention trial conducted by the team, as well as CNBH’s ongoing prevention and clinical services. The previous alcohol prevention trial in the Cherokee Nation, which involved larger high schools in county seats, implemented school-based screening and brief intervention, as well as community organizing to empower citizens to take action to protect youth from easy access to alcohol. Both strategies alone and in combination were successful in reducing alcohol and other drug use among youth (Komro et al., 2015; Komro et al., 2017; Livingston et al., 2018; Wagenaar et al., 2018; Garrett et al., 2019). During this same period, CNBH started the Helping Everyone Reach Out (HERO) Project, the goal of which is to provide a spectrum of effective community-based services and support for children and youth with or at risk of mental and behavioral health challenges. Community organizing was incorporated into the HERO Project as a key strategy to empower and achieve community change.

The population of interest for the current trial, focused on opioid and other drug prevention, includes older adolescents attending high schools in more underserved small-town areas in the 14 counties that partially or fully fall within the Cherokee Nation reservation. The trial aims include implementation and testing of effects of a multilevel integrated preventive intervention through existing structures of CNBH and Oklahoma public schools.

Members from both the CNBH and Emory-based teams agreed that the strongest test of the intervention, using a randomized trial design, would be important to ensure that future prevention efforts were supported by the strongest science. School leadership from 20 high schools agreed to participate and to be randomized to intervention or delayed intervention control condition. Throughout the trial, all participating schools and communities are encouraged to seek resources from the CNBH HERO Project, and are not asked to discontinue or be discouraged from implementing other prevention activities. Following the trial phase of the study, communities assigned to the control condition will be offered prevention strategies found to be effective and will be offered grant writing training and assistance in seeking prevention funds and services provided by the HERO Project. The study design is a cluster randomized trial (CRT) with allocation to study condition, immediate intervention, or delayed intervention control, at the school district/town level. One baseline survey and six follow-up surveys, over a 3.5-year period, will measure patterns of drug use from ages 15 to 17 at baseline to 18 to 20 at final follow-up 6 months following on-time graduation. Intervention activities will begin during the cohort’s 10th grade year and will continue regardless of school status and for 6 months following on-time graduation. The final follow-up survey allows for assessment of changes in drug use outcomes in the important transition from high school to young adulthood.

#### Trial with Urban-Based AI/AN Emerging Adults

The urban-based trial is led by principal investigators Drs. Elizabeth D’Amico (RAND Corporation) and Daniel Dickerson (UCLA, Integrated Substance Abuse Programs (ISAP)) in partnership with Sacred Path Indigenous Wellness Center (SPIWC), a non-profit organization in California that provides substance use and mental health services for AI/AN people. SPIWC provides consultation to ensure that research and services are provided in a culturally appropriate manner and employs AI/AN recruiters and facilitators to assist with community engagement. Dr. Dickerson is Alaska Native (Inupiaq) and the CEO of SPIWC, Dr. Johnson, is Wahpeton Dakota.

The aims of this study are to develop and test a developmentally and culturally appropriate prevention intervention among AI/AN emerging adults. The study plans to enroll 370 participants across the USA who are expected to represent the broader population of 18- to 25-year-old urban AI/AN emerging adults.

This trial builds upon previous work conducted in these communities by this research team for over two decades. For the current trial, we conducted 13 focus groups with AI/AN emerging adults, parents of AI/AN emerging adults, and providers for AI/AN emerging adults to adapt our MICUNAY (Motivational Interviewing and Culture for Urban Native American Youth) program, which was developed for AI/AN urban teens ages 14 to 18 years (Dickerson et al., 2016). Focus groups helped us determine the different challenges that urban AI/AN emerging adults face and led to the development of two culturally appropriate prevention intervention programs that we will be testing in this trial. Participants will be randomized to one of two culturally appropriate interventions, Traditions and Connections for Urban Native Americans (TACUNA), which comprises three workshops and a wellness circle (Dickerson et al., 2022), or an opioid education workshop. Three workshops were chosen for TACUNA based on our previous work to ensure that we could integrate three cultural activities into programming and to provide emerging adults with three opportunities to discuss their social networks. The wellness circle is provided as an additional way for emerging adults to connect with others and broaden their social networks. The opioid education workshop was chosen as a culturally appropriate control condition, and is based on information provided by the National AI/AN Technology and Transfer Center, funded by SAMHSA (Addiction Technology Transfer Center Network, 2021).

Participants will complete surveys at baseline, 3, 6, and 12 months. This research team will compare outcomes to see whether there are differences in opioid, alcohol and cannabis use, spirituality, cultural connectedness, and social networks. This research team will also examine potential mechanisms of change for decreases in substance use outcomes through mediation analyses, including changes in social networks and cultural connectedness. We will test strategies to facilitate sustainability and implementation of TACUNA through key informant interviews and focus groups upon conclusion of the randomized controlled trial. Finally, we will conduct an economic evaluation to quantify programmatic costs and cost-effectiveness of the multitiered intervention approach, relative to opioid education.

### Similarities Between Approaches

#### Integration of Indigenous and Academic Perspectives to Respond to Community Needs

Both trials are the culmination of decades of building trust and partnerships between prevention scientists and AI/AN tribes, organizations, and people to address community needs. The partnership between prevention scientists and CNBH was initiated over a decade ago by the prevention scientists who were motivated to address structural inequities with adaptations of evidence-based prevention strategies and to do so in partnership with a tribal partner. The tribal partners, leadership of CNBH, were motivated to further develop their capacity to implement effective community prevention strategies, as well as to strengthen their research skills. For example, we successfully implemented a community trial in six schools within Cherokee Nation, and these interventions reduced alcohol and other drug use among AI and other youth living in rural at-risk underserved communities (Komro et al., 2015; Komro, Livingston et al., 2017; Livingston, Komro et al., 2018; Wagenaar et al., 2018; Garrett et al., 2019). In preparation for designing the new trial, we conducted in-depth interviews with school leaders and field staff from the previous trial, as well as Cherokee youth and adults. A consistent theme across all interviews was the importance of expanding prevention efforts beyond solely a focus on alcohol, and to comprehensively address mental health and other drugs of concern, including opioid misuse, cannabis, and vaping. School leaders described the ever-changing landscape of drugs of concern (e.g., proliferation of vaping and cannabis products). Cherokee youth and adults shared their concern that it was very easy for youth to get alcohol and other drugs from social sources, including older peers, siblings, and from home. They expressed concerns about family struggles with unemployment and housing. They shared that young people often use substances as a mental health coping strategy or to fit in with their peers. Lessons learned from the first trial and ongoing experiences of CNBH practitioners, as well as in-depth feedback from interviews, were used to guide intervention strategies designed to address individual and community needs. The opioid funding opportunity presented a chance to address opioid use prevention as well as other drugs highlighted by the community.

The RAND/UCLA/SPIWC team of prevention scientists have been working with AI/AN communities for over two decades to develop culturally appropriate programming to address substance use using a community-based participatory approach (Dickerson, Johnson et al., 2012; Dickerson et al., 2014; Dickerson et al., 2016; D'Amico, Dickerson et al., 2020; Dickerson, D'Amico et al., 2021). Our team has conducted two randomized controlled trials in urban communities throughout California to address substance use with teens and adults with positive results (D'Amico, Dickerson et al., 2020; Dickerson, D'Amico et al., 2021).

Similar to the Cherokee Nation trial, we spent the first year of the project working closely with several communities to understand challenges and needs of the community related to substance use as part of the development of TACUNA. We conducted 13 focus groups with AI/AN emerging adults, parents of AI/AN emerging adults, and providers who work with AI/AN emerging adults. Focus groups addressed understanding how to best discuss opioid use, alcohol, and cannabis use among AI/AN emerging adults, talk about social networks and how to make healthy social connections in their urban communities, and what traditional practices are developmentally relevant for 18 to 25 years old. We also worked closely with a Native American graphic artist, Robert Young (Pueblo of Acoma), to design our logo, which the community picked from five different logo options. We meet monthly with our Elder Advisory Board (EAB), made up of key AI/AN community leaders as elders in AI/AN communities hold a revered place as keepers of knowledge. Our EAB collaborates with us to understand how to best identify, reach, and engage urban AI/AN individuals and how to adapt and enhance our programming so that is it developmentally and culturally appropriate. Our team also comprises leaders in the community. Specifically, Dr. Dickerson’s knowledge of traditional practices and involvement within the AI/AN community as an Alaska Native researcher is a key asset to the study and community. Dr. D’Amico’s experience with motivational interviewing (MI) and working with racially/ethnically diverse youth assisted in the integration of traditional practices with MI. Finally, our long-standing collaboration with our community partner, SPIWC, led by Dr. Carrie Johnson has been crucial in ensuring that we are meeting the community’s needs and that research and services are provided in a culturally appropriate manner.

#### Community Partnerships and Engagement

The project in the Cherokee Nation involves four levels of community partnerships and engagement. The first level is the years-long partnership between prevention scientists and CNBH to innovate, implement, and test prevention strategies using rigorous community randomized trial designs. All aspects of the study are designed with input from all team members. The second level of community engagement is with community-based organizations, including 20 Oklahoma public school districts who have signed partnership agreements with our team, and Neighbors Building Neighborhoods Nonprofit Resource Center, which has a long history of partnering with CNBH to provide support and infrastructure for community-based prevention. The third level of community engagement involves high school principals and teachers, community-based organizers, citizen action teams, and other members of the community who will be engaged in the school and community interventions. We strive for ongoing communication and responsiveness to the school and community needs. The fourth and final level of engagement is with youth, as intervention strategies guide and empower young people to make healthy decisions in a supportive environment. All intervention strategies were selected and designed to empower and build capacity among individuals and communities to address needs in a culturally responsive manner.

Similarly, the RAND/UCLA/SPIWC research team has partnered with urban AI/AN communities on various NIH-funded projects that have focused on development and testing of culturally centered substance use treatment and prevention programs for urban AI/AN individuals. The EAB provides oversight of the work through monthly meetings focused on all aspects of the project and helps connect us with the communities. Our work on MICUNAY began because of our prior relationships with urban AI/AN organizations throughout California, established previously with Drs. Dickerson and Carrie Johnson (Dickerson et al., 2016). This work helped to obtain community buy-in for the current trial to analyze the benefits of TACUNA. Our team also works with communities on other projects, including Drum-Assisted Recovery Therapy for Native Americans (Dickerson, D'Amico et al., 2021) and Native American Youth Sleep Health and Wellness (D’Amico, Palimaru et al., 2020; Palimaru et al., 2020), which have continued our partnership and engagement with urban AI/AN populations.

Given the pandemic and need to move to a virtual trial, Drs. Dickerson and Johnson further utilized established partnerships with urban AI/AN organizations across the USA. These partnerships have been strengthened through follow-up reports and meetings with our urban community partners to inform them of preliminary findings.

#### Alignment with Indigenous Cultural Values and Practices: Reinforcing Social Connections

Both interventions emphasize and reinforce positive social connections, reflecting the value placed by Indigenous people on resilience-based strategies to address health challenges. Social connection is at the heart of many AI/AN communities and families. Familial connections are part of how AI/AN people view the world and make decisions. Indeed, empirical research has documented that social support is protective against mental health problems (Wang et al., 2018) and poor psychosocial outcomes among adolescents, including substance use (Heerde & Hemphill, 2018), and is positively associated with resilience among AI adolescents (Stumblingbear-Riddle & Romans, 2012). A literature review of protective factors for AI/AN adolescent health found consistent evidence for the importance of family connectedness, non-familial connectedness, and cultural connectedness (Henson et al., 2017).

Within the urban environment, socially engaging with other AI/AN people and organizations is inherently challenging. This is important to recognize as social disconnectedness has been shown to adversely affect health among ethnic minority groups in urban areas (James et al., 2021). Furthermore, not being able to connect with other AI/AN people and organizations may decrease the potential for enhancing a healthy sense of cultural identity, pride, and learning about and participating in traditional practices.

The prevention approach developed for the Cherokee Nation was guided by the conceptual model based on a socio-ecological and risk and protective informed framework (Hawkins et al., 1992; Keyes et al., 2014; Komro et al., 2016; Wagenaar & Perry, 1994) and an Indigenous relational worldview perspective (Blackstock, 2019; Cross, 2007). A relational worldview emphasizes the importance of balance and complex interrelationships that affect health and well-being and has been represented with a circle representing context, mind, body, and spirit (Cross, 2007). The guiding model for our intervention highlights multilevel and interrelationship of societal, community, social, and individual factors, while considering context, mind, body, and spirit, for drug misuse prevention and mental health promotion among rural and AI youth. Our multilevel intervention seeks to reinforce protective social connections and health-promoting context by combining and enhancing our established interventions, Connect and Communities Mobilizing for Change and Action (CMCA), developed in partnership with the Cherokee Nation and building from CNBH prevention practices and youth services (HERO Project). The Connect intervention focuses on individual-level empowerment to make healthy decisions and also on building social support within the immediate social context, and the CMCA intervention focuses on family and community-level empowerment and building social support and protective, health-enhancing contexts for youth, including both structural and normative dimensions.

The TACUNA intervention utilizes the Northern Plains Medicine Wheel as a culturally acceptable conceptual model routinely used and accepted in tribally diverse populations (Dickerson et al., 2016). The Medicine Wheel symbolizes dimensions of health and the cycles of life (U.S. National Library of Medicine, n.d.). It holds any cultural teachings that address the stages of life, seasons, ceremonial plants, elements of nature, dimensions of well-being, core AI/AN philosophies and values, and additional cultural teachings that may relate to special tribal groups.

TACUNA also provides participants with the opportunity to participate in AI/AN traditional practices. Feedback and suggestions obtained from focus groups highlighted that AI/AN urban emerging adults are very interested in learning more about their culture; however, they also expressed challenges in being able to participate in AI/AN traditional practices within the urban setting. We used feedback obtained from focus groups to match specific AI/AN traditional practices (AI/AN storytelling, cooking, and sage ceremony/spirituality) with content and teaching materials for each workshop utilizing the framework of the Medicine Wheel. At the community level, we provide monthly virtual wellness circles that emerging adults attend and bring members of their social networks. These gatherings help to bring people together to celebrate health and wellness and AI/AN traditions. The wellness circles also focus on how social networks and cultural connectedness influence healthy behaviors.

#### Capacity Building and Empowerment

A goal of both interventions is to build capacity and empower individuals and communities to strengthen self-determination and health. The project in the Cherokee Nation empowers individuals through individual, school, family, community, and media approaches that are informed and led by CNBH and community members. The TACUNA workshops empower emerging young adults to restore cultural identity, pride, and presence through discussion and participation in traditional practices and by providing an opportunity to address the effects of historical trauma experienced by AI/AN people residing in urban areas.

The CNBH Connect intervention is centered around a school-based and universally implemented screening and brief intervention with motivational interviewing (MI), implemented by “Connect coaches” hired by CNBH as family care managers. MI was selected because its non-confrontational style and emphasis on respect and empowerment make it well-suited for adolescents and young adults and particularly AI/AN youth (Gilder et al., 2011). Importantly, we implement Connect universally, to reduce potential stigma associated with speaking to a Connect coach and to reinforce drug-free norms among all students. Universal screening avoids contributing to harmful, negative stereotypes likely to result from intervening with only AI students. Based on formative interviews, Connect coaches will also implement yearly training for teachers, parents, and other community members to help them identify youth with mental health and substance use concerns and develop communication skills to intervene and seek support for those in need (National Council for Mental Wellbeing, 2021).

Communities Mobilizing for Change and Action (CMCA), previously known as Communities Mobilizing for Change on Alcohol, is a community organizing intervention to empower local citizens to address concerns in their communities (Wagenaar & Perry, 1994; Wagenaar et al., 2018). Training and tools are provided to support families and citizens on prevention actions in a culturally responsive manner. Ongoing development of family action kits and action guides—practical evidence-based strategy tools—will be informed with community input to address local needs and context and will include family, school, and community policies and practices.

The TACUNA project is focused on providing culturally appropriate solutions to address the opioid epidemic among AI/AN emerging adults residing in urban areas. Similar to our previous work with urban AI/AN teens, the workshops have a “Pan-Native American” approach that draws upon strengths from different tribal practices and traditions. Furthermore, TACUNA is implemented using an MI approach, and Dr. D’Amico is a member of the Motivational Interviewing Network of Trainers (MINT). Our MICUNAY study was the first to integrate the evidence-based practice of MI with traditional practices for urban AI/AN adolescents (Dickerson et al., 2016; D'Amico, Dickerson et al., 2020), and teens indicated that they felt respected, that the group leader valued their opinion, and that the group leader helped them believe that they could change and improve their life (D'Amico, Dickerson et al., 2020).

Overall, TACUNA empowers individuals through individual, community, and media approaches. First, emerging adults are provided with an opportunity to learn more about their culture, how to make healthy choices around alcohol and other drug use, and ways to increase supportive social network connections. Second, community partners and organizations are empowered to prevent opioid use within their communities, and data generated from this project will be shared at the aggregate level to enhance their knowledge of what is happening in their community. Organizations may also use this information to help with grant funding to further enhance programming within their communities. We also plan to provide trainings on both MI and the protocol for organizations who want to deliver TACUNA within their communities. Because our study has gone virtual due to the pandemic, we have a large social media presence, which has allowed us to bring the program to many different people and organizations across the USA. Finally, our team’s involvement in the HEAL Implementation Science workgroup will help to identify implementation strategies to ensure successful implementation of TACUNA in urban AI/AN communities throughout the USA.

### Differences Between Approaches

#### Implementation Within Complex Cultural Context: Rural Reservation vs Urban Settings

A key difference between the two approaches is that the reservation-based project focuses on one geographic rural region and primarily one tribe, the Cherokee Nation in northeastern Oklahoma, compared with the urban-based project involvement of urban AI/AN emerging adults across the USA who self-identify as AI/AN. A benefit of the reservation-based approach is the ability to tailor interventions to specific cultural values, norms, and practices of the tribe and region. The Cherokee Nation reservation is unique. It transverses communities and school districts across 14 northeastern Oklahoma counties. However, the prevention approaches employed are designed to meet the needs of the whole multicultural region. Therefore, this intervention addresses the diversity that exists within the reservation by building capacity within communities by engaging teachers, families, and other citizens.

TACUNA involves AI/AN emerging adults age 18–25 living in urban areas, and there is great diversity of tribal affiliation as we are involving urban AI/AN communities throughout the USA. For example, Los Angeles is comprised of AI/AN people from over 100 tribes. Therefore, our intervention is designed to provide a more fundamental introduction to AI/AN cultural education and traditional practices. Thus, our “Pan-Native American” approach has the ability to better resonate with a diverse tribal population as opposed to tribal-specific approaches that may typically be taken in reservation/rancheria/village settings.

While both approaches emphasize strengthening social connections, individual capacity, and empowerment, the reservation-based intervention focuses on strategies that directly empower citizens to address context by shaping structural and normative changes within families and communities. The urban-based intervention works to strengthen individual capacity and empowerment to make healthy decisions and increase supportive social networks.

For both projects, the COVID-19 pandemic led to adaptations to implementation approaches which varied given contextual and intervention approaches. In the trial in the Cherokee Nation, implementation delays and then adaptations became necessary due to continued hiring challenges for field staff. For Connect, we shifted to an open-source adaptable computer-based screening and brief intervention program for initial screening and brief intervention. With follow-up by a Connect Coach via Zoom reserved for youth who are demeaned high-risk. The primary adaptation of CMCA was to begin with a focus on family rather than community actions. We are re-developing community action guides into “Family Action Kits” for direct distribution to families via mail and through school and community organizations. Family action kits and corresponding media will move from a focus on home to the larger community over time.

For TACUNA, we had to move all in person group workshops to virtual, and we therefore pilot tested conducting the three workshops virtually with urban AI/AN emerging adults. For example, instead of an in-person cooking demonstration and activity, we mailed cooking ingredients for Three Sisters Stew (dish chosen for cooking) for participants to use at home during the cooking workshop. Participants in the pilot workshop said that having the program virtually made things easier because it did not cost anything in terms of travel time, or paying for gas, and they could still fully engage in all aspects of the program. In addition, the trial was originally supposed to occur only in California; however, when we moved the workshops to virtual, the trial was opened up to all urban AI/AN emerging adults across the USA.

#### Tribal and Elder Oversight

The sovereign status of tribes affords unique rights, including rights in the governance of research. The NIH Tribal Collaboration Working Group of the *All of Us* Research Program Advisory Group is one source that details that sovereign status gives tribes legal rights and privileges that are distinct from racial and ethnic groups (The Tribal Collaboration Working Group, 2018). For the projects described in this paper, tribal oversight of the research is more straightforward with the reservation-based project. The Cherokee Nation Institutional Review Board (CNIRB) serves as the single IRB for the trial and has reviewed and approved the trial protocol. All presentations and manuscripts from the trial will be submitted to the CNIRB for review and approval consideration in accordance with the CNIRB policy. Any entity other than the Cherokee Nation, including its research-intensive partner, Emory University, or the sponsor of the study, wishing to access, view, or use the data from this study must request explicit permission from the Cherokee Nation prior to data access, must agree to abide by all requirements of the Cherokee Nation review process, and must comply with any conditions the Cherokee Nation requires for data access or use.

The RAND/UCLA study recognizes that Indigenous data sovereignty (IDS) emphasizes the right of Indigenous nations and peoples to govern the collection, ownership, and application of its own data (National Congress of American Indians, 2018). The principles of IDS apply on and off tribal lands, pertaining to AI/AN people from sovereign tribes not solely to tribal jurisdictions. As such, any entity wishing to access, view, or use the data from this study must request explicit permission from the TACUNA Urban Intertribal Native American Review Board (UINARB) prior to data access. Any such entity must also agree to abide by all requirements of the UINARB review process and must comply with any conditions the UINARB requires.

## Discussion

Overall, both projects highlight the importance of long-standing relationships with community partners, engaging community at all levels to ensure cultural and developmental appropriateness, and having tribal and elder oversight. These are all key to establishing trust and building confidence in research in these communities and showing how research can benefit AI/AN people. The input of community members ensured that Indigenous cultural strengths and perspectives were integrated across every aspect of the studies. These studies showcase how strong partnerships can advance health and support the conduct of rigorous science to help pinpoint optimal health solutions by identifying efficacious, culturally grounded intervention strategies. Although the sovereign status of tribes demands this type of partnership, this research serves as a model for all community research that has a goal of improving health.

Both projects are also unique and timely and address important needs. For example, the multilevel intervention in the Cherokee Nation is being developed and implemented in small rural communities and populations that are often underserved. The intervention was designed to provide resources, build skills, and empower local people to make changes within their communities to protect young people from the risks associated with mental health challenges and drug use. TACUNA provides urban AI/AN emerging adults with an opportunity to connect virtually with other AI/AN individuals, discuss ways to stay healthy and engage in culture, and find supportive social networks. Many urban AI/AN emerging adults do not have access to cultural resources and support, and TACUNA fills this gap by providing a safe setting in which they can engage with others in their age group and discuss cultural identity in an urban setting. Furthermore, the TACUNA wellness circles offer an opportunity for the community to come together and provide a safe space to connect with culture and share their knowledge. Both projects are being evaluated using rigorous randomized designs at the individual and community levels, using the same outcomes measures. Both are part of the larger HPC with similar implementation and cost analysis strategies. The research partnerships, processes, implementation methods, and trial outcomes of the trials will contribute to the growing prevention science field dedicated to improving Native American health.

Using community-based suggestions, both projects integrate Indigenous perspectives and practices within the prevention strategies and mirror similar approaches used in other health-promoting programs funded under NIH (Crump et al., 2020). Developing culturally centered opioid prevention programs can help address unique issues that AI/AN youth and young adults experience that stem from various historical and intergenerational trauma.

The data ownership and sharing strategies in these projects comport with tribal sovereignty and further provide an example of how research data can be handled in a way that respects the desires of research participants. The approvals required for dissemination of research results will ensure that reports move beyond stigmatizing characterizations to more accurately reflect the strength and resiliencies of Indigenous people. Further they will aid in ensuring that substance use is placed in its full context, moving beyond models that emphasize the role of individual factors, and recognizing the numerous structural factors over which an individual has no control that confer risk. This shift away from stigmatizing portrayals is critical to achieving health equity and developing solutions that have significant impact.

Overall, these studies represent an important contribution to the field and provide critical knowledge on efficacious intervention strategies for a population that has too long been left behind in research. Findings from these large randomized controlled trials can identify developmentally and culturally appropriate strategies to increase resilience among both rural and urban AI/AN adolescents and emerging adults.

## References

[CR1] Addiction Technology Transfer Center Network. (2021). *National American Indian and Alaska Native Addiction Technology Transfer Center (ATTC) Network*. https://attcnetwork.org/centers/national-american-indian-and-alaska-native-attc/home

[CR2] Ahmad, F. B., Rossen, L. M., Sutton, P. (2021). Provisional drug overdose death counts. *National Center for Health Statistics, 2021*. https://www.cdc.gov/nchs/nvss/vsrr/drug-overdose-data.htm

[CR3] Allen J., Mohatt, G. V., Rasmus S. M., Hazel K., Thomas L., Lindley S., & People Awakening Team (2006). The tools to understand: Community as co-researcher on culture specific protective factors for Alaska natives. Journal of Prevention and Intervention in the Community.

[CR4] Allen J, Rasmus SM, Fok CCT, Charles B, Henry D (2018). Multi-level cultural intervention for the prevention of suicide and alcohol use risk with Alaska Native youth: A nonrandomized comparison of treatment intensity. Prevention Science.

[CR5] Blackstock C (2019). Revisiting the breath of life theory. British Journal of Social Work.

[CR6] Blue Bird Jernigan V., D’Amico E.J. (2020). Multilevel and community-level interventions with Native Americans: Challenges and opportunities. Prevention Science.

[CR7] Cross, T. (2007). Through indigenous eyes: Rethinking theory and practice. 2007 Secretariat of National Aboriginal and Islander Child Care (SNAICC), Adelaide, Australia. https://www.snaicc.org.au/through-indigenous-eyes-rethinking-theory-and-practice-keynote-address-2007-cross-t-snaicc-conf-2007-2/

[CR8] Cross, T., Earle, K., Echo-Hawk Solie, H., & Manes, K. (2000). Cultural strengths and chalenges in implementing a system of care model in American Indian communities. *Systems of Care: Promising Practices in Children's Mental Health, 2000 Series, Volume I*. Washington, DC: Center for Efective Colaboration and Practice, American Institutes for Research.

[CR9] Crump AD, Etz K, Arroyo JA, Hemberger N, Srinivasan S (2020). Accelerating and strengthening Native American health research through a collaborative NIH initiative. Prevention Science.

[CR10] D’Amico EJ, Dickerson DL, Brown RA, Johnson CL, Klein DJ, Agniel D (2020). Motivational interviewing and culture for urban Native American youth (MICUNAY): A randomized controlled trial. Journal of Substance Abuse Treatment.

[CR11] D’Amico, E. J., Palimaru, A. I., Dickerson, D. L., Dong, L., Brown, R. A., Johnson, C. L., Klein, D. J., & Troxel, W. M. (2020b). Examining risk and resilience factors in urban American Indian/Alaska Native youth during the coronavirus pandemic. *American Indian Culture and Research Journal*, 44(2), 21–48. 10.17953/aicrj.44.2.d’amico PMC9205322 35719739

[CR12] Dickerson, D. L., D’Amico, E. J., Palimaru, A., Brown, R., Kennedy, D., Johnson, C. L., & Schweigman, K. (2022). Traditions and Connections for Urban Native Americans (TACUNA): Utilizing community-based input in the development of an opioid prevention intervention for urban American Indian/Alaska Native emerging adults. *Journal of Substance Abuse Treatment*, in press. 10.1016/j.jsat.2022.108764 PMC9187599 35450751

[CR13] Dickerson D, Baldwin JA, Belcourt A, Belone L, Gittelsohn J, Keawe’aimoku Kaholokula, J., Lowe, J., Patten, C. A., & Wallerstein, N.  (2020). Encompassing cultural contexts within scientific research methodologies in the development of health promotion interventions. Prevention Science.

[CR14] Dickerson DL, Brown RA, Johnson CL, Schweigman K, D’Amico EJ (2016). Integrating motivational interviewing and traditional practices to address alcohol and drug use among urban American Indian/Alaska Native youth. Journal of Substance Abuse Treatment.

[CR15] Dickerson DL, D’Amico EJ, Klein DJ, Johnson CL, Hale B, Ye F, Dominguez BX (2021). Drum-assisted recovery therapy for Native Americans (DARTNA): Results from a feasibility randomized controlled trial. Journal of Substance Abuse Treatment.

[CR16] Dickerson, D. L., Johnson, C. L., Castro, C., Naswood, E., & Leon, J. M. P. (2012). Community voices: Integrating traditional healing services for urban American Indians/Alaska Natives in Los Angeles County. *Learning collaborative summary report*, 1–42.

[CR17] Dickerson DL, Parker J, Johnson CL, Brown RA, D’Amico EJ (2021). Recruitment and retention in randomized controlled trials with urban American Indian/Alaska Native adolescents: Challenges and lessons learned. Clinical Trials.

[CR18] Dickerson DL, Venner KL, Duran B, Annon JJ, Hale B, Funmaker G (2014). Drum-Assisted Recovery Therapy for Native Americans (DARTNA): Results from a pretest and focus groups. American Indian and Alaska Native Mental Health Research.

[CR19] Duran, E., & Duran, B. (1995). *Native American Postcolonial Psychology*. SUNY Press.

[CR20] Garrett BA, Komro KA, Merlo LJ, Livingston BJ, Rentmeester S, Tobler A, Livingston MD, Kominsky TK (2019). CONNECT: Implementation of a school-based alcohol screening and brief intervention for youth in the Cherokee Nation. Journal of School Health.

[CR21] Gilder DA, Luna JA, Calac D, Moore RS, Monti PM, Ehlers CL (2011). Acceptability of the use of motivational interviewing to reduce underage drinking in a Native American community. Substance Use & Misuse.

[CR22] Gone JP (2012). Indigenous traditional knowledge and substance abuse treatment outcomes: The problem of efficacy evaluation. American Journal of Drug and Alcohol Abuse.

[CR23] Hawkins JD, Catalano RF, Miller JY (1992). Risk and protective factors for alcohol and other drug problems in adolescence and early adulthood: Implications for substance abuse prevention. Psychological Bulletin.

[CR24] Heerde JA, Hemphill SA (2018). Examination of associations between informal help-seeking behavior, social support, and adolescent psychosocial outcomes: A meta-analysis. Developmental Review: DR.

[CR25] Henson M, Sabo S, Trujillo A, Teufel-Shone N (2017). Identifying protective factors to promote health in American Indian and Alaska Native adolescents: A literature review. Journal of Primary Prevention.

[CR26] James C, Corman H, Noonan K, Reichman NE, Jimenez ME (2021). Adolescent chronic health conditions and school disconnectedness. Journal of Developmental & Behavioral Pediatrics.

[CR27] Kaholokula JK, Look M, Mabellos T, Ahn HJ, Choi SY, Sinclair KA, Wills TA, Seto TB, de Silva M (2021). A cultural dance program improves hypertension control and cardiovascular disease risk in Native Hawaiians: A randomized controlled trial. Annals of Behavioral Medicine.

[CR28] Keyes KM, Cerdá M, Brady JE, Havens JR, Galea S (2014). Understanding the rural–urban differences in nonmedical prescription opioid use and abuse in the United States. American Journal of Public Health.

[CR29] Komro KA, Livingston MD, Garrett BA, Boyd ML (2016). Similarities in the etiology of alcohol use among Native American and non-Native young women. Journal of Studies on Alcohol and Drugs.

[CR30] Komro, K. A., Livingston, M. D., Wagenaar, A. C., Kominsky, T. K., Pettigrew, D. W., Garrett, B. A., & Cherokee Nation Prevention Trial Team. (2017). Multilevel prevention trial of alcohol use among American Indian and white high school students in the Cherokee Nation. *American Journal of Public Health*, *107*(3), 453–459. 10.2105/AJPH.2016.303603 10.2105/AJPH.2016.303603 PMC5296689 28103073

[CR31] Komro KA, Wagenaar AC, Boyd M, Boyd BJ, Kominsky T, Pettigrew D, Tobler AL, Lynne-Landsman SD, Livingston MD, Livingston B, Molina MMM (2015). Prevention trial in the Cherokee Nation: Design of a randomized community trial. Prevention Science.

[CR32] Livingston, M. D., Komro, K. A., Wagenaar, A. C., Kominsky, T. K., Pettigrew, D. W., & Garrett, B. A. (2018). Effects of alcohol interventions on other drug use in the Cherokee Nation. *American Journal of Public Health*, *108*(2), 259–261. 10.2105/AJPH.2017.304188 10.2105/AJPH.2017.304188 PMC5846594 29267057

[CR33] Mashpee Wampanoag Tribe. (2021). The 360 project – Turning lives around. Retrieved August 5, 2021 from https://mashpeewampanoagtribe-nsn.gov/opioid-education

[CR34] Meit M, Knudson A (2017). Leveraging interest to decrease rural health disparities in the United States. American Journal of Public Health.

[CR35] National Congress of American Indians. (2018). The National Congress of American Indians Resolution #KAN-18–011: Support of US Indigenous Data Sovereignty and Inclusion of Tribes in the Development of Tribal Data Governance Principles. Retrieved on August 5, 2021 from https://www.ncai.org/attachments/Resolution_gbuJbEHWpkOgcwCICRtgMJHMsUNofqYvuMSnzLFzOdxBlMlRjij_KAN-18-011%20Final.pdf

[CR36] National Council for Mental Wellbeing. (2021). About MHFA. Retrieved on April 29, 2021 from https://www.mentalhealthfirstaid.org/about/

[CR37] Norris, T., Vines, P. L., & Hoeffel, E. M. (2012). The American Indian and Alaska Native Population: 2010. *2010 Census Briefs*. Retrieved on August 6, 2021 from https://www.census.gov/history/pdf/c2010br-10.pdf

[CR38] Palimaru AI, Brown RA, Troxel WM, Dickerson DL, Johnson CL, D’Amico EJ (2020). Understanding sleep facilitators, barriers, and cultural dimensions in Native American urban youth. Sleep Health.

[CR39] Palombi LC, St Hill CA, Lipsky MS, Swanoski MT, Lutfiyya MN (2018). A scoping review of opioid misuse in the rural United States. Annals of Epidemiology.

[CR40] Rasmus, S. M. (2014). Indigenizing CBPR: Evaluation of a community-based and participatory research process implementation of the Elluam Tungiinun (towards wellness) program in Alaska. *American Journal of Community Psychology, 54*(1–2), 170–179. 10.1007/s10464-014-9653-3 10.1007/s10464-014-9653-3 PMC4119544 24756887

[CR41] Rasmus SM, Trickett E, Charles B, John S, Allen J (2019). The qasgiq model as an indigenous intervention: Using the cultural logic of contexts to build protective factors for Alaska Native suicide and alcohol misuse prevention. Cultural Diversity & Ethnic Minority Psychology.

[CR42] Sequist TD (2017). Urgent action needed on health inequities among American Indians and Alaska Natives. The Lancet.

[CR43] Snijder M, Lees B, Stearne A, Ward J, Garlick Bock S, Newton N, Stapinski L (2021). An ecological model of drug and alcohol use and related harms among Aboriginal and Torres Strait Islander Australians: A systematic review of the literature. Preventive Medicine Reports.

[CR44] Soto, C., West, A., Unger, J., et al. (2019). Addressing the opioid crisis in American Indian & Alaska Native communities in California: A statewide needs assessment. Retrieved on August 6, 2021 from https://ipr.usc.edu/wp-content/uploads/2019/11/USC_AI_Report.pdf

[CR45] Stanley LR, Crabtree MA, Swaim RC (2021). Opioid misuse among American Indian adolescents. American Journal of Public Health.

[CR46] Stanley LR, Swaim RC, Smith JK, Conner BT (2020). Early onset of cannabis use and alcohol intoxication predicts prescription drug misuse in American Indian and non-American Indian adolescents living on or near reservations. American Journal of Drug and Alcohol Abuse.

[CR47] Stumblingbear-Riddle G, Romans JSC (2012). Resilience among urban American Indian adolescents: Exploration into the role of culture, self-esteem, subjective well-being, and social support. American Indian and Alaska Native Mental Health Research.

[CR48] Substance Abuse and Mental Health Services Administration. (2021). Tribal opioid response grants. Retrieved on June 18, 2021 from https://www.samhsa.gov/grants/grant-announcements/ti-20-011

[CR49] The Tribal Collaboration Working Group. (2018). Considerations for meaningful collaboration with tribal populations. All of Us Research Program Advisory Panel. https://allofus.nih.gov/sites/default/files/tribal_collab_work_group_rept.pdf

[CR50] Tipps RT, Buzzard GT, McDougall JA (2018). The opioid epidemic in Indian country. Journal of Law, Medicine & Ethics.

[CR51] U.S. National Library of Medicine. (n.d.). Medicine ways: Traditional healers and healing. National Institutes of Health, Health & Human Services: Native Voices. https://www.nlm.nih.gov/nativevoices/exhibition/healing-ways/medicine-ways/medicine-wheel.html

[CR52] Wagenaar AC, Perry CL (1994). Community strategies for the reduction of youth drinking: Theory and application. Journal of Research on Adolescence.

[CR53] Wagenaar AC, Livingston MD, Pettigrew DW, Kominsky TK, Komro KA (2018). Communities mobilizing for change on alcohol (CMCA): Secondary analyses of a randomized controlled trial showing effects of community organizing on alcohol acquisition by youth in the Cherokee nation. Addiction.

[CR54] Wang J, Mann F, Lloyd-Evans B, Ma R, Johnson S (2018). Associations between loneliness and perceived social support and outcomes of mental health problems: A systematic review. BMC Psychiatry.

[CR55] Whitbeck L. B. (2006). *Some guiding assumptions and a theoretical model for developing culturally specific preventions with Nation American people*. Sociology Department, Faculty Publications, 27, University of Nebraska. Accessed 7 March 2022 from https://digitalcommons.unl.edu/sociologyfacpub/27

[CR56] Whitbeck LB, Walls ML, Welch ML (2012). Substance abuse prevention in American Indian and Alaska Native communities. American Journal of Drug and Alcohol Abuse.

[CR57] Whitesell, N. R., Mousseau, A., Parker, M., Rasmus, S., & Allen, J. (2020). Promising practices for promoting health equity through rigorous intervention science with Indigenous communities. *Prevention Science, 21*(Suppl 1), 5–12. 10.1007/s11121-018-0954-x 10.1007/s11121-018-0954-x PMC6778005 30443847

[CR58] Wilson, N., Kariisa, M., Seth, P., Smith, H., 4th, & Davis, N. L. (2020). Drug and opioid-involved overdose deaths - United States, 2017–2018. *MMWR. Morbidity and Mortality Weekly Report, 69*(11), 290–297. Accessed 29 April 2021 from 10.15585/mmwr.mm6911a4external 10.15585/mmwr.mm6911a4 PMC7739981 32191688

[CR59] Witko, T. M. (2006). *Mental health care for urban Indians: Clinical insights from Native practitioners*. American Psychological Association.

